# Whole-Brain Mapping of the Expression Pattern of *T1R2*, a Subunit Specific to the Sweet Taste Receptor

**DOI:** 10.3389/fnana.2021.751839

**Published:** 2021-10-28

**Authors:** Jea Hwa Jang, Ha Kyeong Kim, Dong Woo Seo, Su Young Ki, Soonhong Park, Sang-Hyun Choi, Dong-Hoon Kim, Seok Jun Moon, Yong Taek Jeong

**Affiliations:** ^1^BK21 Graduate Program, Department of Biomedical Sciences, Korea University College of Medicine, Seoul, South Korea; ^2^Department of Pharmacology, Korea University College of Medicine, Seoul, South Korea; ^3^Department of Oral Biology, BK21 PLUS Project, Yonsei University College of Dentistry, Seoul, South Korea

**Keywords:** taste receptors, G-protein coupled receptor, knock-in mouse, immunohistochemistry, neurochemistry

## Abstract

Chemosensory receptors are expressed primarily in sensory organs, but their expression elsewhere can permit ligand detection in other contexts that contribute to survival. The ability of sweet taste receptors to detect natural sugars, sugar alcohols, and artificial sweeteners suggests sweet taste receptors are involved in metabolic regulation in both peripheral organs and in the central nervous system. Our limited knowledge of sweet taste receptor expression in the brain, however, has made it difficult to assess their contribution to metabolic regulation. We, therefore, decided to profile the expression pattern of T1R2, a subunit specific to the sweet taste receptor complex, at the whole-brain level. Using *T1r2-Cre* knock-in mice, we visualized the overall distribution of *Cre*-labeled cells in the brain. *T1r2-Cre* is expressed not only in various populations of neurons, but also in glial populations in the circumventricular organs and in vascular structures in the cortex, thalamus, and striatum. Using immunohistochemistry, we found that *T1r2* is expressed in hypothalamic neurons expressing neuropeptide Y and proopiomelanocortin in arcuate nucleus. It is also co-expressed with a canonical taste signaling molecule in perivascular cells of the median eminence. Our findings indicate that sweet taste receptors have unidentified functions in the brain and suggest that they may be a novel therapeutic target in the central nervous system.

## Introduction

The principal function of chemosensory receptors in the sensory organs is the detection of exogenous chemicals (Yarmolinsky et al., 2009; Chaudhari and Roper, 2010; Liman et al., 2014). In the oral cavity, taste receptors sensitive to sweet, umami, and bitter tasting compounds convert chemical information into biological signaling (Zhao et al., 2003; Mueller et al., 2005). Umami and sweet are detected by T1R1 and T1R2, respectively, along with T1R3, which acts with T1R1 and T1R2 as a co-receptor (Zhao et al., 2003). Bitter tastes, in contrast, are detected by bitter taste receptors, a family of proteins comprising 25 members in humans and 35 members in mice (Mueller et al., 2005).

Chemosensory receptors are also expressed in other organs and tissues where they play unexpected physiological roles responding to their cognate ligands (Calvo and Egan, 2015). For example, bitter taste receptors expressed in human airway smooth muscle respond to their ligands and induce bronchodilation to remove harmful substances from the airways (Deshpande et al., 2010). In addition to defensive roles like this, the extraoral expression patterns of taste receptors often hint at the roles they play in the regulation of metabolism and physiology. For example, in addition to their expression in taste buds, the subunits of the sweet taste receptor complex—T1R2 and T1R3—are expressed in the intestine (Howitt et al., 2020), pancreas (Nakagawa et al., 2009; Kyriazis et al., 2012), brain (Ren et al., 2009; Kohno et al., 2016), and testes (Mosinger et al., 2013). In pancreatic beta cells, sweet taste receptors detect blood fructose, which potentiates the effect of blood glucose in the release of insulin (Kyriazis et al., 2012). Sweet taste receptors are also expressed in the hypothalamus where they mediate the cellular responses to artificial sweeteners (Ren et al., 2009; Kohno et al., 2016; Benford et al., 2017), implying a role for T1R2 and T1R3 in the central regulation of metabolism. Therefore, sweet taste receptors can function as detectors of internal body state.

Several brain areas maintain metabolic homeostasis by responding, not only to circulating hormones such as insulin, leptin, ghrelin, and angiotensin II (Spanswick et al., 1997, 2000; Cowley et al., 2003; Paes-Leme et al., 2018), but also to nutrients (Sohn and Ho, 2020). According to their responses to glucose, glucose-sensing neurons can largely be divided into two groups, glucose-excited (GE) neurons and glucose-inhibited (GI) neurons (Sohn and Ho, 2020). Like pancreatic beta cells, GE neurons detect glucose primarily via glucose transporter 2 (GLUT2), glucokinase, and ATP-sensitive potassium channel (K_ATP_) (Ashford et al., 1990; Miki et al., 2001; Jordan et al., 2010), but its incompleteness has raised other glucosensing mechanisms. Recently, sweet taste receptor has been proposed to be an alternative low affinity glucose sensor in the brain (Ren et al., 2009; Kohno et al., 2016; Benford et al., 2017). However, the lack of the whole brain expression pattern of sweet taste receptor underestimates the importance of its contribution. Therefore, it is crucial to clarify tissue distribution and neurochemical properties of T1R2 to understand the diverse nutrient sensing mechanisms in the brain.

Here, we investigated the expression of T1R2 across the whole brain. We generated knock-in mice expressing Cre recombinase under the control of the *T1r2* promoter to make it easier to characterize the neurochemical properties of *T1r2*-expressing cells. By combining this line with the *ROSA26-LSL-tdTomato* fluorescent marker, we found broad but specialized expression of T1R2 across the brain. In particular, we observed intense fluorescence in hypothalamic nuclei near the ventricles. Using IHC, we found various cell types—including neurons, astrocytes, tanycytes, and perivascular cells—both labeled by *T1r2*-*Cre* and expressing canonical taste signaling molecules. In the arcuate nucleus (ARC), we observed both T1R2-expressing neuropeptide Y (NPY) and proopiomelanocortin (POMC)-expressing neurons. Moreover, in addition to the expected neuronal expression of *T1r2*-*Cre*, we unexpectedly observed *T1r2*-*Cre* labeling of cerebrovascular structures of the forebrain. According to our results, T1R2 is expressed in locations expected to be advantageous in the detection of circulating metabolites and exogenous sweeteners in the brain. Thus, our study not only broadens our understanding of the expression pattern of a single chemosensory receptor in the central nervous system, but we expect it will also contribute a novel target for the development of therapeutic approaches to metabolic regulation.

## Materials and Methods

### Mice

All animal experiments were approved by the Animal Care Committee of Korea University College of Medicine (KOREA-2019-0132). All mice were maintained under standard animal housing conditions [12 h light-dark cycles with *ad libitum* access to normal chow diet (SAFE^®^ A03, France) and water]. The *T1r2* knock-in allele was generated by Macrogen (Korea). The original founder was generated under C57BL6/N background. The donor construct sequences, including the homology arms and the Cre sequence used to achieve homology-mediated direct repair, are indicated in Supplementary Figure 1.

To generate *T1r2*-*tdTomato* mice, *T1r2-Cre* mice were bred to *ROSA26-LSL-tdTomato* mice (JAX007908). To obtain *T1r2*-*tdTomato*:*Npy-hrGFP* or *T1r2*-*tdTomato*:*POMC-hrGFP* mice, *T1r2*-*tdTomato* mice were bred to either of *Npy-hrGFP* (JAX006417) or *POMC-hrGFP* (JAX006421) mice, respectively. We confirmed the genotype of every mouse by PCR amplification of genomic DNA extracted from their tails (MyTaq Extract-PCR kit, BIO-21127, Bioline, United Kingdom). The primer sequences used for genotyping are indicated in Table 1.

**TABLE 1:** Primer sequences used for genotyping.

**Genes**		**Sequences**
*T1r2*	Forward	5′-CAA TGA GGC TGG GCA TCG TCT AAG-3′
	Reverse	5′-CAC CAC TTG CAA CTT GAC TTT GAA CTC-3′
*Cre*	Forward	5′-TCC AAT TTA CTG ACC GTA CAC CAA-3′
	Reverse	5′-CCT GAT CCT GGC AAT TTC GGC TA-3′
*tdTomato*	Forward	5′-CAA CAT GGC CGT CAT CAA AGA-3′
	Reverse	5′-CTT GTA CAG CTC GTC CAT GCC-3′
*POMC-hrGFP*	Forward	5′-TGG CTC AAT GTC CTT CCT GG-3′
	Reverse	5′-GGT GCG GTT GCC GTA CTG GA-3′
*Npy-hrGFP*	Forward	5′-TAT GTG GAC GGG GCA GAA GAT CCA GG-3′
	Reverse	5′-GGT GCG GTT GCC GTA CTG GA-3′

### Two Bottle Tests

Adult mice were acclimated individually in plastic cages with *ad libitum* access to food. Drinking water was supplied via two custom sipper tubes for at least 1 week. After acclimation, most of the mice did not show any position bias. A session was composed of two test days. Mice were allowed to choose between sipper tubes containing water and tastants for 2 days, and the tube positions were switched daily. Each session was separated from the next by a 5-day interval to prevent the results of later sessions from being affected by previous sessions. When identical tastants were being tested, they were presented in increasing concentrations. The volume of ingested liquid was measured and a preference index (P.I.) was calculated according to the following equation: P.I. = volume of ingested tastant solution/(volume of ingested pure water + volume of ingested tastant solution). The numbers of animals subjected to two bottle test are four and three for wild-type and *T1r2* KO, respectively.

### Immunohistochemistry

*T1r2-tdTomato* mice were serially perfused with 0.1 M phosphate buffered saline (PBS) and 4% paraformaldehyde (PFA) in PBS. Their brains were dissected from their skulls, post-fixed overnight, and cryoprotected in 30% sucrose in PBS at 4°C for several days until sinking. They were then embedded in Tissue-Tek OCT (Sakura, Japan). Cryoblocks were stored at −80°C until used. Brain samples were cut into coronal sections with a thickness of 30 μm. Every third free-floating section from the anterior olfactory bulb to the caudal cerebellum was collected. Tongue samples were cut into coronal sections with a thickness of 12 μm and directly attached to slide glass. The sections were then blocked in 5% goat or donkey serum in 0.2% Triton X-100 PBS (PBST) for 30 mins at room temperature. Primary antibodies were diluted in the corresponding blocking buffer and incubated overnight. After washing three times with PBST for 10 mins each, they were incubated in secondary antibodies dissolved in PBST for 2 h at room temperature. DAPI stain (1:5,000; D9542, Sigma) was added after three washes and the sections were mounted with Vectashield (Vector Laboratories, Burlingame, CA, United States) and a cover glass. All the primary and secondary antibodies used are listed in Table 2. Images were acquired with either an LSM 700 or LSM800 confocal microscope (Zeiss, Germany). Total 14 male and 2 female mice were subjected to immunostaining, and we could not find any sexual differences on expression pattern.

**TABLE 2:** The lists of primary and secondary antibodies used in this study.

	**Source**	**Cat#**	**Titer**
**Primary antibodies**			
Goat anti-tdTomato	Sicgen	AB8181-200	1:1,000
Rabbit anti-DsRed	Clontech	632496	1:1,000
Rabbit anti-NeuN	Abcam	Ab177487	1:1,000
Mouse anti-GFAP	Sigma	G3893	1:400
Goat anti-IBA1	Novus Biological	NB100-1028	1:400
Chicken anti-MBP	Invitrogen	PA1-10008	1:400
Guinea pig anti-TRPM5	This study	N/A	1:1,000
Rabbit anti-PLCβ2	Santa Cruz	sc-206	1:1,000
Goat anti-GNAT3	Aviva System	OAEB00418	1:2,500
**Secondary antibodies**			
Donkey anti-rabbit Alexa 488	Invitrogen	A32790	1:1,000
Donkey anti-goat Alexa 488	Invitrogen	A11055	1:1,000
Donkey anti-rabbit Alexa 555	Invitrogen	A31572	1:1,000
Donkey anti-goat Alexa 555	Invitrogen	A32816	1:1,000
Goat anti-chicken Alexa 488	Invitrogen	A32931	1:1,000
Goat anti-mouse Alexa 488	Invitrogen	A32723	1:1,000
Goat anti-guinea pig Alexa 488	Invitrogen	A11073	1:1,000
Goat anti-rabbit Alexa 555	Invitrogen	A32732	1:1,000

### Co-localization Analysis

Every third brain slice covering the ARC was collected and subjected to a double IHC staining protocol. Co-localization analysis was performed with Imaris (Bitplane, Zurich, Switzerland). To determine whether hrGFP fluorescent signals showed co-localization with tdTomato fluorescent signals, the merged images were analyzed with the Imaris co-localization module. Spots were assigned to specific labels based on peak mean intensities. A co-localization threshold of 0.6 μm was set as the pixel boundary occupied by a single parenchymal cell.

### FITC-Dextran Perfusion

Total 2 mL of FITC-Dextran 70 kDa (5 mg/mL, Sigma, 46945) was injected into the left ventricle of deeply anesthetized *T1r2*-*tdTomato* mice at 2 mL/min. Brain was harvested immediately after perfusion, post-fixed in 4% PFA for 24 h, and immersed in 30% sucrose in PBS overnight. Following procedures were conducted similarly with conventional IHC methods.

### Statistical Analyses

All data are expressed as means ± S.E.M. Statistical differences among groups were analyzed using two-sample *t*-tests. Asterisks indicate ^∗^*P* < 0.05, ^∗∗^*P* < 0.01, and ^∗∗∗^*P* < 0.005.

## Results

### Generation of a *T1r2-Cre* Knock-In Strain

**FIGURE 1:**
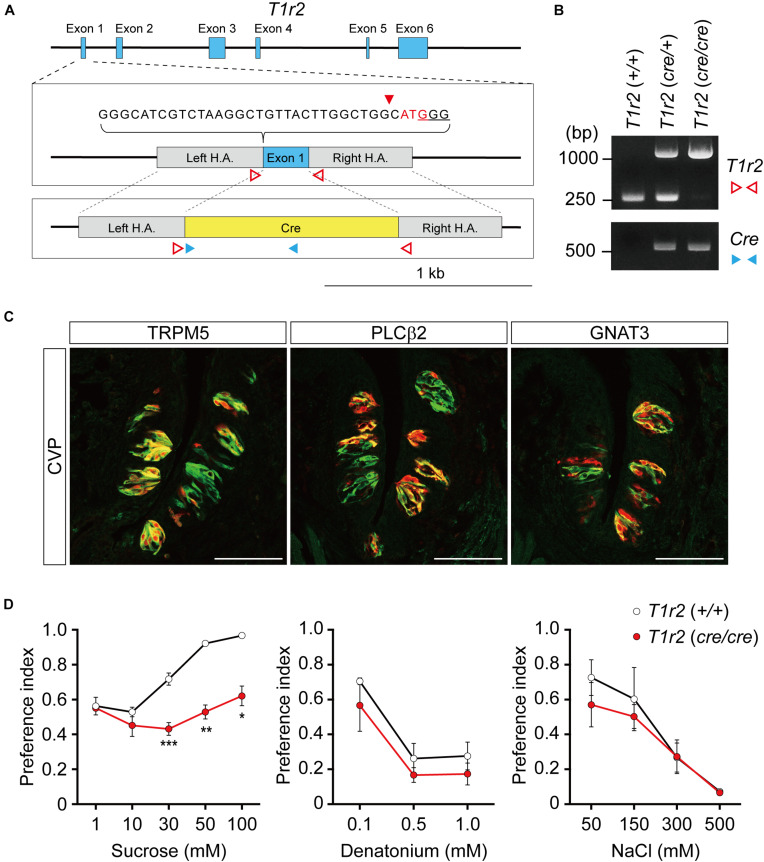
*T1r2-Cre* mouse strain generation and transgene expression analysis. **(A)** *T1r2* locus schematic and the targeting constructs for *T1R2-Cre*. The genomic DNA sequences of the gRNA target are indicated. The PAM sequence used for the gRNA is underlined and the cleavage site is indicated by the red triangle. The *T1r2* start codon appears in red, and the blue boxes indicate *T1r2* exons. The colored triangles indicate the primers used for genomic DNA PCR: *T1r2* (red blank); *Cre* (blue). **(B)** Confirmation of precise genome editing with genomic DNA PCR analysis using the primer pairs indicated in panel **(A)**. The predicted amplicon sizes are 261 and 1,030 bp for the wild-type and mutant alleles *of T1r2*, respectively, and 584 bp for the Cre transgene. **(C)** Co-localization of tdTomato with type II taste cell markers in circumvallate papillae (CVP). **(D)** Confirmation of the *T1r2* knock-out phenotype using two bottle tests. Data are presented as means ± S.E.M. Two-sample *t*-tests were performed for statistical analysis. *n* = 5 (wild-type) and 3 (knock-out), respectively. *P < 0.05, **P < 0.01, and ***P < 0.005.

To genetically label *T1r2-*expressing cells, we generated *T1r2-Cre* knock-in mice using the CRISPR/Cas9 system. By substituting the coding region of the first exon of *T1r2* with Cre sequence using homology-directed repair, we were able to generate mice that express Cre recombinase under the control of the endogenous promoter and enhancers of the *T1r2* gene (Figure 1A). We confirmed the precise genomic exchange for the target region by genomic PCR (Figure 1B). We designed this mutant allele for use, not only as a *Cre* driver for the *T1r2* gene in the heterozygous state, but also as a *T1r2* knock-out (KO) in the homozygous state.

To confirm its function, we first performed immunohistochemistry (IHC) staining of the tongue. After crossing *T1r2*-*Cre* mice to *ROSA26-LSL-tdTomato* mice, we visualized *Cre*-labeled cells in their progeny (hereafter, *T1r2-tdTomato*). In the tongue, we found tdTomato fluorescence restricted to taste bud-containing papillae (Figure 1C). In circumvallate papillae (CVP), *T1r2*-expressing cells belong to the subset of TRPM5 and PLCβ2-expressing cells. This indicates *T1r2*-*Cre* labels type II taste receptor cells (TRCs). The fluorescence we observed, however, also showed only a partial overlap with GNAT3 (Figure 1C). Given that GNAT3 is expressed preferentially in bitter TRCs in CVP (Kim et al., 2003; Kusakabe et al., 2005; Sainz et al., 2007; Shindo et al., 2008; Tizzano et al., 2008), our data suggest *T1r2*-*Cre* accurately recapitulates the expression of native *T1r2* in sweet TRCs. Next, by performing two-bottle behavioral assays, we found homozygous mice showed impaired choice behaviors toward sucrose but not denatonium or NaCl (Figure 1D). This is consistent with previous studies that used other mutant *T1r2* alleles (Zhao et al., 2003). Thus, our data justify the use of this novel strain in further studies.

### Distribution of *T1r2-Cre* Expressing Cells in the Brain

**FIGURE 2:**
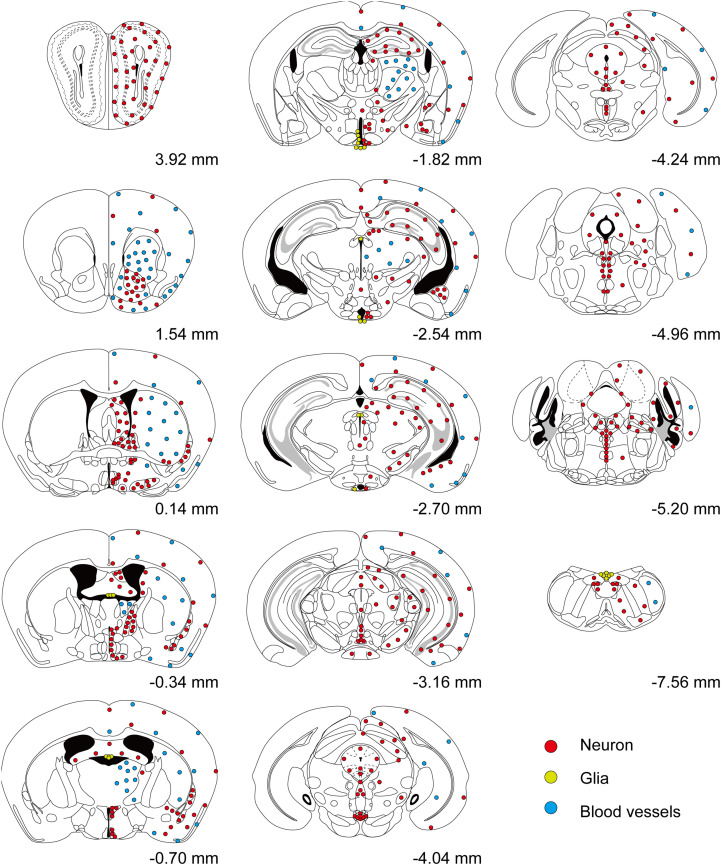
Overall distribution of *T1r2-Cre*-labeled cells in the brain. Coronal section figures were modified from the Paxinos mouse brain atlas (third edition) and re-drawn. Relative positions compared to bregma are indicated. Red circles (neurons); yellow circles (astrocytes); and blue circles (blood vessels).

**FIGURE 3:**
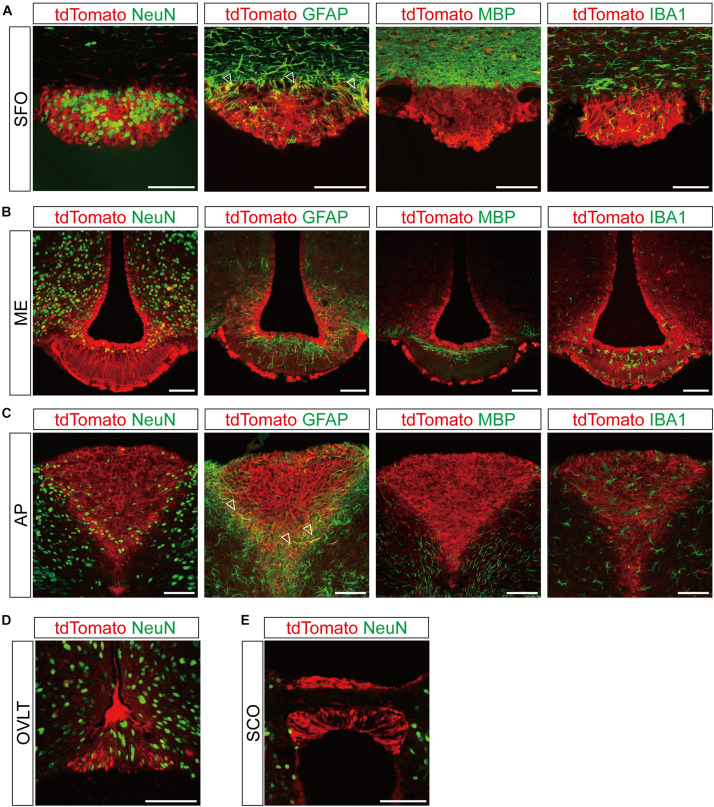
Representative confocal images showing tdTomato fluorescence in the circumventricular organs (CVOs) of *T1r2*-tdTomato mice. **(A)** Subfornical organ (SFO), **(B)** median eminence (ME), **(C)** area postrema (AP), **(D)** organum vasculosum lamina terminalis (OVLT), and **(E)** subcommissural organ (SCO). Representative markers for neurons (neuronal nuclei, NeuN), astrocytes (glial fibrillary acidic protein, GFAP), oligodendrocytes (myelin basic protein, MBP), and microglia (ionized calcium-binding adaptor molecule, IBA1) are indicated in green; tdTomato is indicated in red. White triangles indicate the overlap of the green and red signals. Scale bar, 100 μm.

**FIGURE 4:**
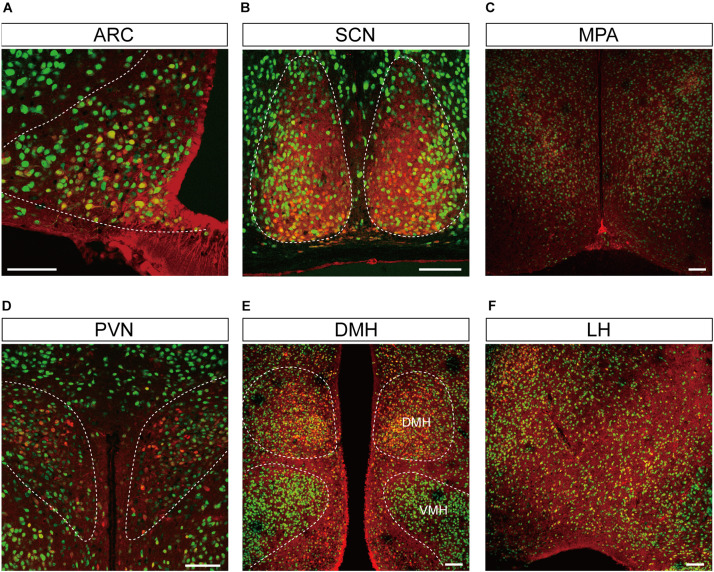
Representative confocal images showing tdTomato fluorescence in the hypothalamic nuclei of *T1r2*-tdTomato mice. **(A)** Arcuate nucleus (ARC), **(B)** suprachiasmatic nucleus (SCN), **(C)** median preoptic area (MPA), **(D)** paraventricular nucleus (PVN), **(E)** dorsomedial hypothalamic nucleus (DMH), and **(F)** Lateral hypothalmus (LH). Anti-NeuN signals (green); tdTomato (red). Scale bar, 100 μm.

**FIGURE 5:**
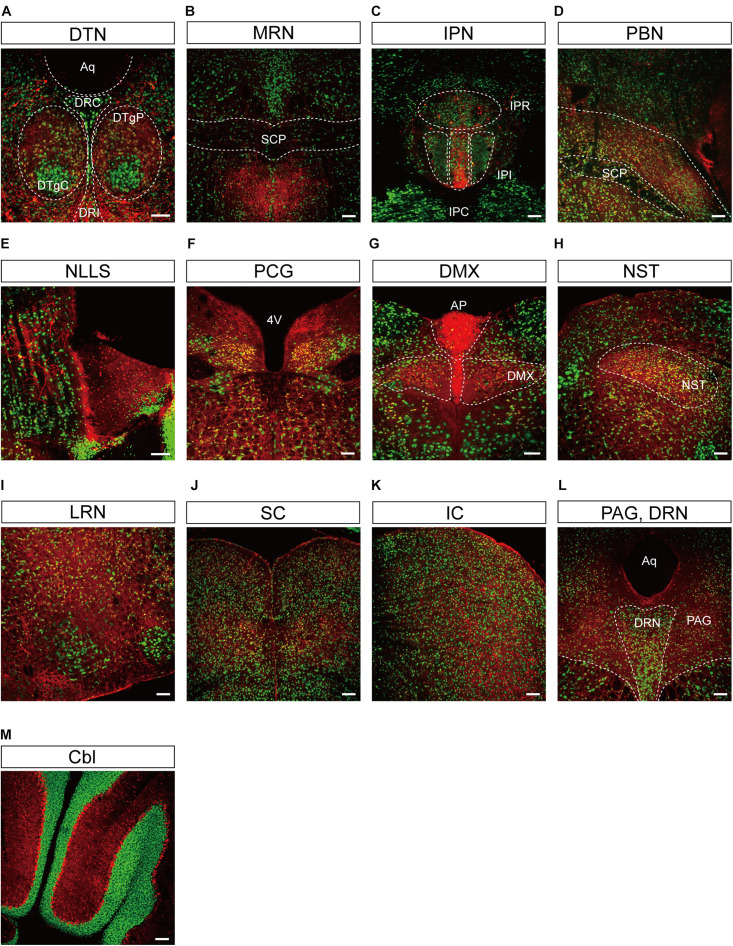
Representative confocal images showing tdTomato fluorescence in the midbrain, hindbrain, and cerebellum of *T1r2*-tdTomato mice. **(A)** Dorsal tegmental nucleus (DTN). The caudal (DRC), interfascicular (DRI), central (DTgC), and pericentral parts (DTgP) are marked. Aqueduct (Aq), **(B)** the median raphe nucleus (MRN) and superior cerebellar peduncles (SCP) are indicated, **(C)** interpeduncular nucleus (IPN). The rostral (IPR), caudal (IPC), and intermediate subnucleus (IPI), **(D)** parabrachial nucleus (PBN), **(E)** nucleus of the lateral lemniscus (NLLS), **(F)** pontine central grey (PCG). 4^th^ ventricle (4V), **(G)** AP and dorsal motor nucleus of the vagus nerve (DMX), **(H)** nucleus of the solitary tract (NST), **(I)** lateral reticular nucleus (LRN), **(J)** superior colliculus (SC), **(K)** inferior colliculus (IC), **(L)** periaqueductal gray (PAG) and dorsal raphe nucleus (DRN), and **(M)** Cerebellum (Cbl). White dotted lines indicate the border of nearby areas. Anti-NeuN signals (green); tdTomato (red). Scale bar, 100 μm.

**FIGURE 6:**
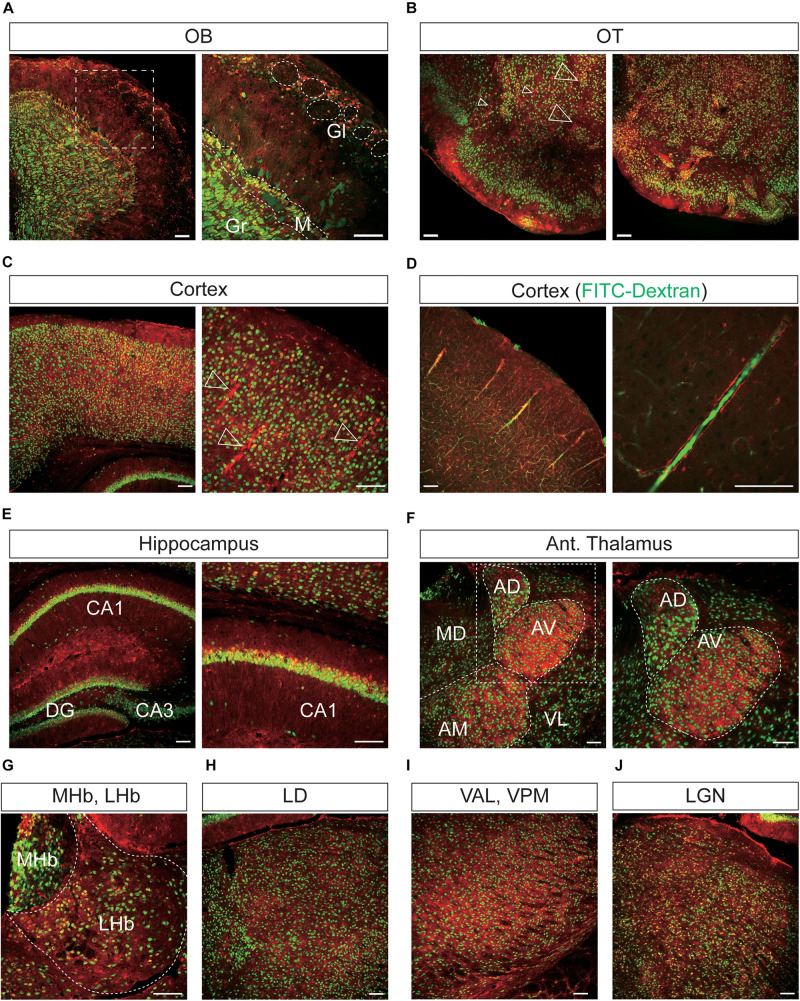
Representative confocal images showing tdTomato fluorescence in the olfactory bulb, cerebral cortex, and thalamus of *T1r2*-tdTomato mice. **(A)** Olfactory bulb (OB). Glomeruli (Gl), mitral layer (M), and granular layer (Gr) are indicated with white dotted lines, **(B)** olfactory tubercle (OT), **(C)** cortex, **(D)** cortex. FITC-Dextran (green). **(E)** In the hippocampus, Cornu Ammonis area 1 (CA1), CA3, and the dentate gyrus (DG) are labeled. **(F)** Anterior thalamus. *Left*: Anterodorsal (AD), anteroventral (AV), anteromedial (AM), mediodorsal (MD), and ventrolateral (VL) nuclei are labeled; *Right*: magnified images of the white dotted box in the left image, **(G)** medial (MHb) and lateral habenula (LHb), **(H)** laterodorsal nucleus (LD) of the thalamus, **(I)** ventroanterolateral (VAL) and ventroposterior medial (VPM) nuclei of the thalamus, and **(J)** lateral geniculate nucleus (LGN). White dotted lines indicate the borders of nearby areas. White empty triangles indicate vascular structures. Anti-NeuN signals [green except for panel **(D)**]; tdTomato (red). Scale bar, 100 μm.

**FIGURE 7:**
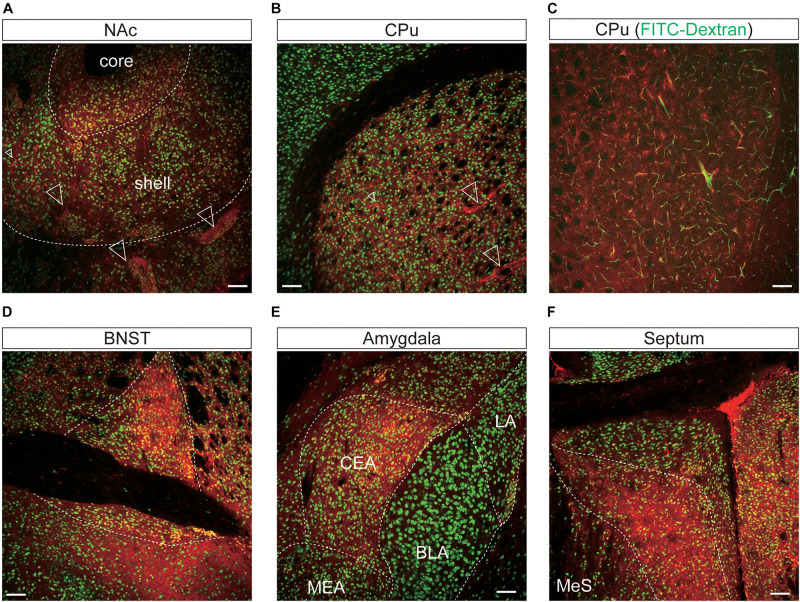
Representative confocal images showing tdTomato fluorescence in the mesolimbic pathways of *T1r2*-tdTomato mice. **(A)** Nucleus accumbens (NAc). The core and shell of the NAc are indicated, **(B)** caudate putamen (CPu), **(C)** caudate putamen. FITC-Dextran (green). **(D)** Bed nucleus of the stria terminalis (BNST). **(E)** In the amygdala, the central amygdala (CEA), medial amygdala (MEA), lateral amygdala (LA), and basolateral amygdala (BLA) are indicated. **(F)** Septal nuclei. White dotted lines indicate the borders of nearby areas. White empty triangles indicate vascular structures. Anti-NeuN signals [green except for panel **(C)**]; tdTomato (red). Scale bar, 100 μm.

We inspected the brains of *T1r2-tdTomato* mice from the olfactory bulb to the brain stem. The overall distribution and intensity of tdTomato fluorescence are indicated in Figure 2 and Table 3, respectively. Every brain section was subjected to double IHC against tdTomato and NeuN to recognize the overall structure. Most tdTomato-expression overlapped with anti-NeuN staining, especially in the olfactory bulb (OB), cerebral cortex, thalamus, nucleus accumbens (NAc), bed nucleus of the stria terminalis (BNST), amygdala, and septal nuclei. This overlap also included several hypothalamic nuclei, including the medial preoptic area (MPA), paraventricular nucleus (PVN), suprachiasmatic nucleus (SCN), ARC, lateral hypothalamic nucleus (LH), and dorsomedial hypothalamic nucleus (DMH) (Figures 2–7 and Table 3).

**TABLE 3:** Anatomic mapping of *T1r2*-expressing brain area.

**Region**	***T1r2***	**Neuron**	**Glia**	**Vessel**
**Telencephalon**				
Olfactory bulb				
Glomerular layer	++	O		
External Plexiform and Mitral cell layer	++	O		
Internal Plexiform and Granule cell layer	+++	O		
Olfactory tubercle	++	O		O
**Nucleus accumbens**				
Shell	+	O		
Core	++	O		
Claustrum	+	O		
**Lateral Septum**				
Intermediate	+	O		
Dorsal	+	O		
Ventral	++	O		
Subfornical organ	++++		O	
Cortex		O		
**Isocortex**				
Somatomotor areas	+	O		O
Somatosensory areas	+	O		O
Gustatory areas	+	O		O
Visceral areas	+	O		O
Auditory areas	+	O		O
Visual areas	+	O		O
Anterior cingulate areas	+	O		O
Prelimbic area	+			O
Infralimbic area	+			O
Orbital area	+			O
Agranular insular area	+			O
Retrosplenial area	+	O		O
Posterior parietal association areas	+	O		O
Temporal association areas	+	O		O
Perirhinal area	+	O		O
Ectorhinal area	+	O		O
**Olfactory areas**				
Anterior olfactory nucleus	++	O		
Tenia tecta	+	O		O
Piriform area	+			O
Nucleus of the lateral olfactory tract	+	O		O
Cortical amygdala nucleus	+	O		O
Piriform-amygdala area	+			O
Postpiriform transition area	+	O		O
Bed nucleus of the stria terminalis	+++	O		
Preoptic area	+	O		

**Hippocampus**				

**CA3**				
Rostral	+	O		
Caudal	+	O		
CA2	+	O		
CA1 (Caudal)	+	O		
Dentate gyrus	++	O		
Fasciola cinerea	+	O		
Indusium griseum	+	O		
**Amygdala**				
Central Amygdala	+++	O		
Medial Amygdala	+	O		
Basolateral Amygdala	–	X		
Basomedial Amygdala	+	O		
Lateral Amygdala	–	X		
Organum vasculosum of the lamina terminalis	++	O		

**Diencephalon**				

**Thalamus**				
Anterior thalamic nuclei	+++			O
Ventral posteromedial thalamic nuclei	+			O
Paraventricular nucleus	+			O
Lateral dorsal nucleus	++			O
**Geniculate nucleus**				
Dorsolateral	+	O		
Pregeniculate	+	O		
Medial	+	O		
Nucleus reuniens	+	O		
Ventral anterior thalamic nucleus	++			O
Parafascicular thalamic nucleus	+			O
Subcommissural organ	++		O	
Precommissural nucleus	+		O	
Ventromedial thalamic nucleus	+			O
Medial habenular nucleus	++	O		
Lateral habenular nucleus	+	O		
Thalamic reticular formation	+			O
**Hypothalamus**				
Anterior hypothalamus	–	X		
Dorsomedial hypothalamus	++	O		
Ventromedial hypothalamus	–	X		
Paraventricular nucleus	++	O		
Arcuate nucleus	++++	O		
Lateral hypothalamus	+	O		
Posterior hypothalamus	+	O		
Suprachiasmatic nucleus	++++	O		
Retrochiasmatic nucleus	–	X		
Zona incerta	+	O		
Ventromedial hypothalamus	–	X		
**Mesencephalon**				
Periaqueductal gray	+	O		
Dorsal nucleus raphe	+	O		
Interpenduncular nucleus	+++	O		
Median raphe nucleus	+++	O		
Premamillary nucleus	+	O		
Mammillary nucleus	+	O		
Pretectal nucleus	+	O		
Superior colliculus	+	O		
Inferior colliculus	+	O		
Dorsal tegmental nucleus	+++	O		
Ventral tegmental area	+	O		
Parabrachial nucleus	+	O		
**Brainstem**				
Area postrema	++++	X	O	
Nucleus tractus solitarius	+	O		
Pontine central gray	++	O		
Dorsal motor nucleus of the vagus nerve	+	O		
Nucleus of the lateral lemniscus	+	O		
Hypoglossal nucleus	+	O		
Nucleus of Roller	–	X		
Reticular formation	+	O		
Medullary reticular nucleus	+	O		
Intermediate reticular nucleus	+	O		
Spinal nucleus of the trigeminal	+	O		O
**Cerebellum**				
Molecular layer	+	O		
Purkinje layer	+++	O		
Granule cell layer	++	O		

Although we did observe tdTomato fluorescence scattered throughout the brain, there were significant concentrations in specific regions. The most prominent signals were located near the ventricular system (Figure 3 and Table 3), with intense staining in the circumventricular organs (CVOs)—the subfornical organ (SFO), the median eminence (ME), the area postrema (AP), the organum vasculosum lamina terminalis (OVLT), and the subcommissural organ (SCO) (Figure 3). There was also a morphologically heterogenous population of tdTomato-positive cells in the mediobasal hypothalamus—not only parenchymal cells with a neuron-like morphology, but also cells lining the internal wall of the third ventricle and the external wall of the ME (Figures 3, 4). The tdTomato-positive cells in the SCN and MPA, in contrast, were morphologically homogenous (Figure 4). In addition, while the dorsal hypothalamus, PVN, DMH, and LH showed significant tdTomato fluorescence, other important hypothalamic nuclei, including the anterior hypothalamic nucleus and ventromedial hypothalamic nucleus did not (Figure 4).

We also observed significant tdTomato expression in the hindbrain (Figure 5). The dorsal tegmental nucleus (DTN), median raphe nucleus (MRN), and interpeduncular nucleus (IPN) each showed a bright and densely fibrous arbor of staining arising from a relatively limited number of tdTomato-expressing cells (Figures 5A–C). In addition, we observed some tdTomato labeling in the parabrachial nucleus (PBN), the nucleus of the lateral lemniscus (NLLS), the pontine central grey (PCG), the dorsal motor nucleus of the vagus nerve (DMX), the nucleus of the solitary tract (NST), and the reticular nucleus (RN) (Figures 5D–I). In the midbrain, we observed labeling in a subset of neurons in the superior (SC) and inferior colliculi (IC), as well as in the ventrolateral PAG and DRNs (Figures 5J–L). In the cerebellum, we observed mutually exclusive expression of either tdTomato or NeuN, indicating expression of T1R2 in Purkinje cells rather than granular cells (Figure 5M).

In the OB, the tdTomato and anti-NeuN signals showed overlap outside the olfactory glomeruli and in the mitral and granular layers, indicating that periglomerular cells, mitral cells, and granular cells express *T1r2* (Figure 6A). When we examined the cortex, we found most cortical regions showed some scattered fluorescence. Although some tdTomato-expressing cells showed overlap with anti-NeuN staining, most did not (Figures 6B,C). In the hippocampus, neurons in CA1, CA2, CA3, and dentate gyrus (DG) were tdTomato-positive regardless of their rostrocaudal position (Figure 6E). In the anterior thalamus, the tdTomato signal was concentrated in the anterodorsal (AD), anteroventral (AV), and anteromedial (AM) nuclei, as well as in the lateral habenula (Figures 6F,G). In contrast, we observed significant staining scattered throughout the entire posterior thalamus (Figures 6H–J).

In the mesolimbic system, the lateral septum (LS), NAc, caudate putamen (CPu), BNST, central (CEA) and median amygdala (MEA), and septal nuclei expressed tdTomato in some neurons (Figure 7), but the ventral tegmental area (VTA) did not.

### Vascular Expression of *T1r2-Cre*

We found that the many *T1r2*-expressing cells in the cortex, thalamus, and striatum are negative for anti-NeuN. Moreover, we noticed that most of the non-neuronal *T1r2*-expressing cells have a vascular-like luminal structure (Figures 6B,C,F,H,I, 7A,B, white empty triangles). These presumed blood vessels appear to be of various sizes, implying a general *T1r2* expression in the cerebrovascular system. Thick vessels run straight, finally branching into several thin ones (Figures 6B,C, 7A,B, white empty triangles). The thinnest vessels comprised single layers of cells, interconnected with one another to form a vascular web (Figures 6B,C,F,H,I, 7A,B, white empty triangles). To confirm whether the tubular structures formed by *T1r2*-expressing cells are blood vessels, we visualized blood vessels directly by transcardial injection of FITC-dextran (70 kDa) and found the spatial proximity between FITC signals and *T1r2*-expressing cells in cortex and CPu (Figures 6D, 7C). We also observed pericytic tdTomato expression distributed homogenously throughout the cortex, posterior thalamus, and dorsal striatum, as well as more prominent expression in the anterodorsal (AD), anteroventral (AV), and anteromedial (AM) nuclei of the anterolateral thalamus (Figures 6, 7). These data indicate an enrichment of *T1r2* in the neurovascular system.

### Glial Expression of *T1r2-Cre* in Circumventricular Organs

Across the entire brain, CVOs showed such strong tdTomato fluorescence that we could detect it without immunostaining. When we performed double IHC with anti-NeuN, however, we found that not all tdTomato-expressing cells in the CVOs were neurons (Figure 3). To identify their cellular identities, we next conducted double IHC with the following glial cell markers: Glial Fibrillary Acidic Protein (GFAP) for astrocytes; Ionized Calcium-Binding Adaptor molecule (IBA1) for microglia; and Myelin Basic Protein (MBP) for oligodendrocytes.

In the dorsal periphery of the SFO, a subset of tdTomato-expressing cells were positive for GFAP (Figure 3A). Due to the restricted subcellular localization of GFAP protein in astrocytic processes rather than cell bodies, we found that the cytosolic tdTomato signal showed only partial overlap with anti-GFAP signal in each labeled cell. There was, however, no overlap with anti-IBA1 or anti-MBP (Figure 3A). Similarly, the ventrolateral margin of the AP showed co-expression of tdTomato and anti-GFAP (Figure 3C), indicating *T1r2* is expressed in astrocytes in the SFO and AP.

The labeled cells in the ARC-ME had heterogenous morphology owing to a heterogeneity of their cellular identities. In the ARC-ME complex parenchyma, tdTomato-expressing cells showed a neuron-like morphology and expressed NeuN (Figures 3B, 4A). The cells surrounding the wall of the third ventricle, however, expressed neither neuronal nor glial markers (Figure 3B). Instead, we were able to identify these cells as tanycytes by their long processes projecting radially from the ventricular wall to the parenchyma. We also observed strong fluorescence signal in the perivascular cells covering the ventral margin of the ME (Figure 3B). In addition, we observed tdTomato expression in the cells of the OVLT at the anterior end of the third ventricle and in the SCO at the posterior end of the third ventricle (Figures 3D,E).

### Expression of *T1r2-Cre* in Hypothalamic Neuropeptide Y/Agouti-Related Peptide and Proopiomelanocortin Neurons

**FIGURE 8:**
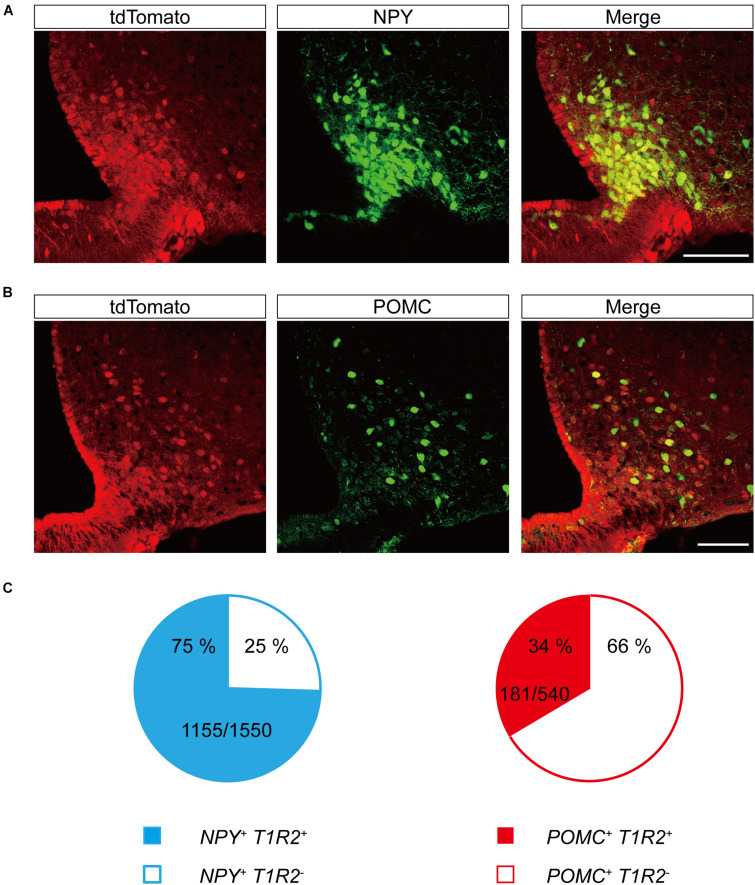
Representative confocal images showing tdTomato fluorescence in hypothalamic neuropeptide Y (NPY) or proopiomelanocortin (POMC)-expressing neurons of *T1r2*-tdTomato mice. **(A)** Co-expression of tdTomato with *Npy-hrGFP*. **(B)** Co-expression of tdTomato with *POMC-hrGFP*. Anti-GFP signals (green), and tdTomato (red). Scale bar, 100 μm. **(C)** Quantification of hypothalamic NPY neurons (blue) and POMC neurons (red) that co-express tdTomato. Filled and blank areas indicate the presence and absence of tdTomato expression, respectively.

The primary centers for central metabolic regulation in the ARC-ME complex are bimodal (Sohn et al., 2013). Depletion of energy is detected by NPY/Agouti-related peptide (AgRP)-expressing neurons (Betley et al., 2015; Beutler et al., 2017; Su et al., 2017), whereas storage of energy is detected by POMC-expressing neurons (Beutler et al., 2017). To determine whether the neurons expressing *T1r2* are NPY/AgRP neurons, POMC neurons, or both, we generated triple transgenic mice expressing *T1r2*-*Cre* and *Rosa-LSL-tdTomato*, along with either *Npy*-*hrGFP* or *POMC*-*hrGFP*. Most of the parenchymal tdTomato signal we observed showed overlap with NPY-expressing neurons (Figure 8A). In contrast, only a small number of tdTomato-labeled neurons were also positive for POMC (Figure 8B). Together, we observed tdTomato fluorescence in 75% (1,155 of 1,550 cells) of *Npy* positive neurons and 34% (181 of 540 cells) of POMC positive neurons (Figure 8C). These data suggest *T1r2* is expressed both in hypothalamic NPY/AgRP and POMC neurons.

### Co-localization of Canonical Taste Signaling Molecules

In taste buds, type II TRCs employ proteins like GNAT3, PLCβ2, and TRPM5 downstream of taste receptor signaling (McLaughlin et al., 1992; Wong et al., 1996; Perez et al., 2002; Liu and Liman, 2003; Zhang et al., 2003; Mueller et al., 2005). We wondered whether such canonical taste signaling molecules are also co-expressed with T1R2 in the brain. Thus, we again conducted double IHC experiments to visualize PLCβ2, GNAT3, or TRPM5 expression alongside tdTomato expression.

**FIGURE 9:**
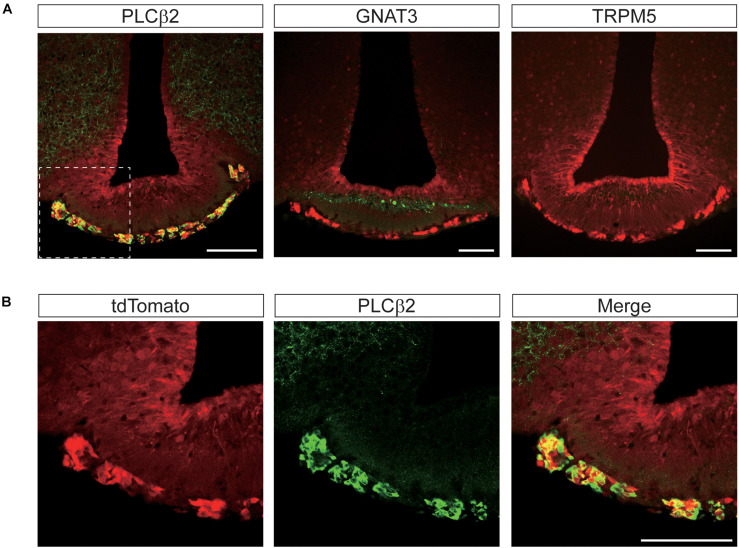
Representative confocal images showing tdTomato fluorescence in the perivascular cells of the median eminence (ME) in *T1r2*-tdTomato mice. **(A)** Double immunostaining of the ME for tdTomato and some canonical taste signaling molecules. Phospholipase Cβ2 (PLCβ2), α-gustducin (GNAT3), and transient receptor potential M5 (TRPM5). **(B)** Magnified view of the white dotted box in Figure 8A. Representative markers are green; tdTomato appears in red. Scale bar, 100 μm.

Remarkably, we found complete co-localization of PLCβ2 with tdTomato in hypothalamic perivascular cells of the leptomeningeal layer, but not in other areas (Figure 9A). Although anti-GNAT3 marked the median eminence, it did not co-localize with tdTomato (Figure 9A). We also found that none of the brain regions that expressed tdTomato were stained with the antibody against TRPM5 (Figure 9A). Given that the specificity of the antibodies we used was already validated in taste buds (Figure 1C), the relative lack of GNAT3, PLCβ2, and TRPM5 in the central nervous system indicates a true scarcity.

## Discussion

In this study, we have described the comprehensive distribution of T1R2 across the whole brain using genetically engineered *Cre*-expressing mice. Moreover, we have revealed a detailed neurochemical characterization of many T1R2-expressing cells using double IHC experiments that labeled both Cre-driven fluorescence in combination with markers for specific neuronal and non-neuronal cell populations. We found that *T1r2*-expressing cells are distributed widely throughout the brain but especially concentrated near the ventricles. *T1r2* is expressed in neurons, as previous studies suggested, but we unexpectedly discovered significant expression in cerebrovascular structures as well. This particular location for the *T1r2*-expressing cells provides them with access to circulating nutrient (e.g., sugars) in both the blood and brain simultaneously, suggesting a role for sweet taste receptors in the regulation of metabolic homeostasis.

Several researchers have reported the existence of glucose-sensing brain neurons that express T1R2 (Ren et al., 2009; Kohno et al., 2016). Most GE neurons detect glucose by metabolizing it. Briefly, glucose is taken up by GLUT2 and phosphorylated by glucokinase to produce ATP. The resulting increase in ATP induces the closure of the Kir6.2 channel, ultimately leading to neuronal depolarization (Ashford et al., 1990; Miki et al., 2001; Jordan et al., 2010). There are other GE neurons that are sensitive to much higher concentrations of glucose and that are thus referred to as high glucose-excited (HGE) neurons (Fioramonti et al., 2004). Diverse molecular sensors—sodium-glucose transporter 1 (SGLT1), SGLT3, and sweet taste receptors—have been proposed as the candidate mechanism of underlying the unique sensitivity of HGE neurons (Yang et al., 1999; Diez-Sampedro et al., 2003; O’Malley et al., 2006; Ren et al., 2009; Kohno et al., 2016). Especially, the low affinity of sweet taste receptors for glucose may simply explain glucose concentration-dependent selectivity (Zhao et al., 2003; Treesukosol et al., 2011).

Through a series of physiology experiments, Kohno et al. (2016) predicted the expression of sweet taste receptors in the ARC based on responses to the artificial sweetener sucralose (Kohno et al., 2016; Benford et al., 2017). They found sucralose activates a small population of hypothalamic POMC neurons that also responds to high concentrations of glucose, indicating that they are HGE neurons (Kohno et al., 2016). Thus, Kohno et al. suggested sweet taste receptors may serve as glucose sensors in HGE neurons. Our genetic labeling experiments partly support their prediction. The ratio of POMC^+^ neurons to sucralose responsive neurons (15%) they identified was similar to the ratio of POMC^+^, T1R2^+^ neurons to POMC^+^ neurons we observed in this study (34%). The subtle discrepancy seems to be due to the low number of POMC neurons recorded in the previous study (Kohno et al., 2016).

Most G-protein coupled receptors (GPCRs) can couple to multiple Gα proteins, as well as b-arrestin, depending on the context of the cell in which they are expressed (Kroeze et al., 2015; Olsen et al., 2020). Taste receptors, which belong to the GPCR superfamily, generally couple to GNAT3 in type II taste cells (McLaughlin et al., 1992; Zhang et al., 2003; Mueller et al., 2005). But not all type II taste cells express GNAT3 (Kim et al., 2003; Kusakabe et al., 2005; Sainz et al., 2007; Shindo et al., 2008; Tizzano et al., 2008). Rather than GNAT3, the sweet taste receptor-expressing cells of foliate and circumvallate papillae express Gα14, suggesting sweet taste receptors use Gα14 as an effector (Kim et al., 2003; Kusakabe et al., 2005; Sainz et al., 2007; Shindo et al., 2008; Tizzano et al., 2008). Thus, just as the leptin and insulin receptors function differently in hypothalamic POMC and NPY/AgRP neurons, it seems T1R2 may also employ distinct modes of action in different cell types. Indeed, the rare co-expression of canonical taste signaling molecules in most *T1r2*-expressing cells in the brain implies that the sweet taste receptors in the brain employ downstream signaling pathways distinct from those employed by the same receptors in type II taste bud cells. It is also possible that a biased agonism of T1R2 is what directs the choice of distinct intracellular signal transduction pathways depending on the ligand that activates the sweet receptor.

We expect T1R2 is expressed in the brain because it is important for some physiological function, not only in the ARC, but also in other brain regions. For example, T1R2-expressing cells in the SFO and AP may detect elevated glucose levels in the cerebrospinal fluid, inducing diabetic thirst and nausea, respectively. Alternatively, sweet taste receptors may be involved in the entrainment of circadian rhythms in the SCN in response to circadian glucose fluctuations. Indeed, a previous study using Ca^2+^ imaging of brain slices that covered the ARC revealed that tanycytes respond to sucralose (Benford et al., 2017). Tanycytes, pericytes, and perivascular cells regulate the permeability of the blood brain barrier (BBB) (Langlet et al., 2013). Thus, sweet taste receptors may prove to be useful targets for drugs aimed at manipulating BBB permeability.

What role do sweet taste receptors play in the brain? Can the *T1r2*-expressing cells we identified in this study detect metabolites like glucose? The novel mouse strain we established in this study will be useful in clarifying the function of T1R2 and the cells that express it in the brain. We will explore T1R2’s function using the strain in a homozygous state as a T1R2 KO. We will also manipulate the activity of *T1r2-*expressing cells using our strain in the heterozygous state as a Cre driver in combination with optogenetic and chemogenetic tools. Soon, we hope to clarify the physiology of taste receptors in the brain, providing novel insights for drug discovery and various therapeutic interventions.

## Data Availability Statement

The original contributions presented in the study are included in the article/Supplementary Material, further inquiries can be directed to the corresponding authors.

## Ethics Statement

The animal study was reviewed and approved by Animal Care Committee of Korea University College of Medicine.

## Author Contributions

JJ and YJ conceptualized and designed the research. JJ and HK conducted the immunohistochemistry experiments and acquired confocal microscopic images. JJ conducted genomic DNA PCR and behavioral experiments. DS quantified the number of hrGFP-expressing neurons that co-express tdTomato. SK conducted the tongue histology experiments. SP, SM, and YJ designed the mutant constructs. S-HC, D-HK, SM, and YJ analyzed and interpreted the data. YJ supervised the project and wrote the manuscript. All authors read and approved the final manuscript.

## Conflict of Interest

The authors declare that the research was conducted in the absence of any commercial or financial relationships that could be construed as a potential conflict of interest.

## Publisher’s Note

All claims expressed in this article are solely those of the authors and do not necessarily represent those of their affiliated organizations, or those of the publisher, the editors and the reviewers. Any product that may be evaluated in this article, or claim that may be made by its manufacturer, is not guaranteed or endorsed by the publisher.
