# Supplementary material for: Whole-Brain Mapping of the Expression Pattern of T1R2, a Subunit Specific to the Sweet Taste Receptor
Source: Front Neuroanat. 2021 Oct 28;15:751839. doi: 10.3389/fnana.2021.751839 (PMC8581048; doi:10.3389/fnana.2021.751839)
Supplement: Supplementary file 1 [file Data_Sheet_1.DOCX]

##### Supplemental Information

Whole-brain mapping of the expression pattern of T1R2, a subunit specific to the sweet taste receptor

Jea Hwa Jang, Ha Kyeong Kim, Dong Woo Seo, Su Young Ki, Soonhong Park, Sang-Hyun Choi, Dong-Hoon Kim, Seok Jun Moon, and Yong Taek Jeong

ACCAGACAGAAGACATACAAGAGGGTATAGGGGTCACAAGGGCACATCGAGGAAGGTGGTTGGGAAAGGAGCTTGGGATACCCCAGTCTCCTCTACCCATTGGGTGGGTGGGGGCAGCTCTGGGCTGCATACCAGGTGTATGCCAAGGGAGACACCAAATGAATGGATGAGGCTGGGCTCACTGTGACCATGGCTGTCTCCTCTGCCTGCCTTTGTGTGACCACCATGGCTGACTGCCACCTTCACAGCCAATGGCCACACAGGTATGAGCTCAGGCAGAAGGCTGGGTGCAGCGACGTAAAAAATTGCATGTTCTCCCAAGCCATGCAGGCCCCACTGGGCCCCACAAATATATCCTGCTCCAGTCTCTGTTCCTTGCCTGGAGCCCTTAAGGATGTCCGCTCATCCCCGGGCAGGATCGTCACTATGTAAATGTCTGTGCAAATGCTTCAGTGTGGGGTGACCGGCTCCACAATGAGGCTGGGCATCGTCTAAGGCT*G****G****TAC****C***TGGCATGGCCAATTTACTGACCGTACACCAAAATTTGCCTGCATTACCGGTCGATGCAACGAGTGATGAGGTTCGCAAGAACCTGATGGACATGTTCAGGGATCGCCAGGCGTTTTCTGAGCATACCTGGAAAATGCTTCTGTCCGTTTGCCGGTCGTGGGCGGCATGGTGCAAGTTGAATAACCGGAAATGGTTTCCCGCAGAACCTGAAGATGTTCGCGATTATCTTCTATATCTTCAGGCGCGCGGTCTGGCAGTAAAAACTATCCAGCAACATTTGGGCCAGCTAAACATGCTTCATCGTCGGTCCGGGCTGCCACGACCAAGTGACAGCAATGCTGTTTCACTGGTTATGCGGCGGATCCGAAAAGAAAACGTTGATGCCGGTGAACGTGCAAAACAGGCTCTAGCGTTCGAACGCACTGATTTCGACCAGGTTCGTTCACTCATGGAAAATAGCGATCGCTGCCAGGATATACGTAATCTGGCATTTCTGGGGATTGCTTATAACACCCTGTTACGTATAGCCGAAATTGCCAGGATCAGGGTTAAAGATATCTCACGTACTGACGGTGGGAGAATGTTAATCCATATTGGCAGAACGAAAACGCTGGTTAGCACCGCAGGTGTAGAGAAGGCACTTAGCCTGGGGGTAACTAAACTGGTCGAGCGATGGATTTCCGTCTCTGGTGTAGCTGATGATCCGAATAACTACCTGTTTTGCCGGGTCAGAAAAAATGGTGTTGCCGCGCCATCTGCCACCAGCCAGCTATCAACTCGCGCCCTGGAAGGGATTTTTGAAGCAACTCATCGATTGATTTACGGCGCTAAGGATGACTCTGGTCAGAGATACCTGGCCTGGTCTGGACACAGTGCCCGTGTCGGAGCCGCGCGAGATATGGCCCGCGCTGGAGTTTCAATACCGGAGATCATGCAAGCTGGTGGCTGGACCAATGTAAATATTGTCATGAACTATATCCGTAACCTGGATAGTGAAACAGGGGCAATGGTGCGCCTGCTGGAAGATGGCGATTGAGTGAGTTCAAAGTCAAGTTGCAAGTGGTGGTGCAGGGGTGGGGCGGAGGTCAGGACCCTGGAAAGTGCCCAGAATGCATCTCTTCAGTCATTGCCAAACCTCCAGTCCTAGATCCAGCCTGCTAGCCCCAGTGCCCCCTGCACGATCTGTGCCAACCCCCAATCCTAGATCCAGCCTGCTAGCCCTCAGTGCCCCTCCCCTTGCACAATCTGTTGAAATTTTAAGTGGTTGTTTGCAACCCAGAGACCTGGTTTGTCCCTAAGTTACATCCCTGGAAAGACAGGGTGTTTAAACTTACAGAGTCACCATCAAAGCCATCCTCAGCCACTCGGGCAAATGGGGGAGCCCCACCCCCAATAAGACCATGTCAAAGCCTTGTCTAGACATCAGGCTCTGCCTTACCAGGCAGAAGGTGGTGTTTGGGTAGCAAGCTCTGACACTGGACACTGGAGGGCAGCTTTGCAGGTCCCAAGGATGTGGCTGGGGAGACAGGGAGTGCT

**SUPPLEMENTAL FIGURE 1** Double-strand donor DNA sequence used for genome editing. Cre-encoding sequences are indicated as red color letters. Homology arm and sgRNA target sequences are indicated as gray and yellow color background, respectively. To prevent re-cut of knock-in allele by sgRNA, we generated silent mutation in sgRNA target sequences. The mutated sequences are indicated as bold italic.
